# Hexavalent Chromium Induces Defense Responses, Hepatocellular Apoptosis, and Lipid Metabolism Alterations in New Zealand Rabbit Livers

**DOI:** 10.3390/metabo15100637

**Published:** 2025-09-23

**Authors:** Junzhao Yuan, Lei Zhang, Xiuqing Li, Xinfeng Li, Pandeng Zhao, Xiaoli Ren, Yuzhen Song

**Affiliations:** 1 Zhengzhou Key Laboratory of Animal Nutrition Metabolic and Poisoning Diseases, College of Veterinary Medicine, Henan University of Animal Husbandry and Economy, Zhengzhou 450000, China; 2 College of Veterinary Medicine, Henan University of Animal Husbandry and Economy, Zhengzhou 450000, China; 80773@hnuahe.edu.cn (L.Z.); 241038@hnuahe.edu.cn (X.L.); 80366@hnuahe.edu.cn (P.Z.); 3 Henan Key Laboratory of Unconventional Feed Resources Innovative Utilization, Institute of Livestock Product Quality and Safety Technology Research, Henan University of Animal Husbandry and Economy, Zhengzhou 450046, China; xinfengli@hnuahe.edu.cn

**Keywords:** hexavalent chromium, New Zealand rabbit, liver, antioxidant genes, fat metabolism, trace elements, apoptosis

## Abstract

**Background**: Hexavalent chromium (Cr(VI)) can migrate into soil and water, posing risks to animal health. However, it remains unclear whether Cr(VI) perturbs essential trace elements and antioxidant gene expression, triggers apoptosis, or disrupts hepatic lipid metabolism in New Zealand rabbits. **Methods**: To address this knowledge gap, twenty-four 30-day-old New Zealand rabbits were randomly allocated to one control and three Cr(VI)-treated groups (differing in Cr(VI) concentration) and maintained for 28 days. Livers were then harvested for analysis. Total Cr and essential trace elements were quantified by ICP-OES. Hematoxylin–eosin staining and transmission electron microscopy were employed to assess histopathological and ultrastructural alterations, respectively. Hepatic lipid accumulation was visualized with Oil Red O staining. QRT-PCR was used to determine the expression of antioxidant and lipid-metabolism-related genes. **Results**: Cr(VI) was detectable in liver tissue at all exposure levels and was accompanied by significant decreases in four essential trace elements (Fe, Mn, Zn, and Se); Cu displayed a biphasic response, rising at lower Cr(VI) doses before declining at higher doses. Histopathological and ultrastructural analyses revealed overt hepatic injury. Notably, all Cr(VI) treatments elevated antioxidant gene expression, indicating activation of hepatic defense pathways. Lipid metabolism was also disrupted, evidenced by increased lipid deposition and up-regulation of genes governing hepatic fat metabolism. **Conclusions**: Collectively, these findings demonstrate that Cr(VI) elicits dose-dependent activation of hepatic antioxidant defenses, promotes apoptosis, and induces lipid-metabolic disorders in New Zealand rabbit hepatocytes. This study provides novel mechanistic insights into Cr(VI)-induced hepatotoxicity and offers a valuable reference for evaluating the hepatic risks of environmental Cr(VI) exposure in this species.

## 1. Introduction

Chromium (Cr) is ubiquitously distributed in the environment and occurs predominantly in three valence states: metallic Cr (Cr^0^), trivalent chromium (Cr(III)), and hexavalent chromium (Cr(VI)). Metallic Cr, valued for its exceptional hardness, is primarily employed in stainless-steel production. Biologically active Cr(III), present in natural foods and living organisms, potentiates insulin signaling, fosters protein synthesis, and supports cellular growth and development; it is therefore classified as an essential trace element for mammals [1]. In stark contrast, Cr(VI) is a highly toxic environmental heavy-metal oxyanion, most commonly encountered as chromate (CrO_4_^2−^) or dichromate (Cr_2_O_7_^2−^). Owing to its strong oxidizing capacity, Cr(VI) is extensively used in metallurgy, chemical manufacturing, and refractory materials. Industrial processes such as sodium dichromate (Na_2_Cr_2_O_7_) and chromic anhydride (CrO_3_) production generate large volumes of Cr(VI)-laden wastewater, exhaust gases, and solid residues, which pose serious ecological risks and cause huge economic loss [2,3]. China is the world’s leading consumer of Cr(VI)-bearing ores, processing more than 20 million tons annually. Nationwide, the cumulative area of Cr(VI)-contaminated sites exceeds 2 million m^2^, distributed across over 20 provinces. Geological features, industrial activities, and agricultural practices drive a pronounced Cr(VI) contamination gradient from northeast to southwest; more than 60 such sites have been documented [4]. With respect to soil pollution, Cr ranks fifth in China, surpassed only by cadmium (Cd), mercury (Hg), arsenic (As), and lead (Pb). Agricultural soils exhibit Cr concentrations ranging from 1.48 to 820.24 mg/kg, while Cr ranks second in water pollution after Hg and Cd [5]. Soil Cr(VI) concentrations frequently exceed 10,000 mg/kg—three orders of magnitude above China’s screening limit of 3.0 mg/kg. Groundwater Cr(VI) levels can reach several hundred micrograms per liter, tens of thousands of times higher than the national standard. In some Cr(VI)-ore-affected water bodies, concentrations approach 10 mg/kg. Cr accumulation in soil–plant systems markedly threaten the health of adjacent fauna and human populations [6]. Xu et al. reported that 21.85% of China’s farmland exceeds the national standard for Cr(VI), and elevated levels have been detected in several agricultural commodities [7]. Consequently, Cr(VI) contamination in China warrants urgent scientific attention and comprehensive mitigation efforts.

Studies have demonstrated that Cr(VI) can enter animals via inhalation, ingestion, and dermal absorption, leading to bioaccumulation in multiple species, including the African catfish (*Clarias gariepinus*), Japanese quail (*Coturnix japonica*), and bighead carp (*Hypophthalmichthys nobilis*), with the highest burdens detected in liver and kidney tissues [8,9,10]. Inflammation and oxidative stress are important mechanisms of poisoning [11,12,13]. Controlled laboratory exposures have further shown that Cr(VI) provokes inflammation, reproductive impairment, progressive bioaccumulation, and elevated antioxidant enzyme activities in the tissues of *Channa asiatica*, the mud clam *Geloina erosa*, and Wistar rats [14,15,16]. Worldwide, workers in electroplating, leather tanning, and mining industries chronically exposed to Cr(VI) exhibit increased incidences of respiratory diseases—such as asthma, rhinitis, pulmonary fibrosis, and chronic obstructive pulmonary disease—along with cutaneous ulcers and allergic dermatitis [17,18,19,20]. As a high-valence cation, Cr(VI) is approximately –100-fold more toxic than Cr(III). This heightened toxicity stems from its capacity to deplete intracellular antioxidants (Glutathione and Ascorbic Acid) and antioxidant enzymes (Catalase, Superoxide Dismutase, or Glutathione Peroxidase) while generating transient valence intermediates (Cr(V)• and Cr(IV)). During Cr(VI)-induced oxidative stress, reactive oxygen species (ROS) are generated; these ROS can directly damage DNA and inhibit critical enzymatic activities in animal cells. Consequently, Cr(VI) is more potently carcinogenic than other heavy metals such as Pb, Cd, or Hg [21]. Beyond apoptosis, Cr(VI) can trigger alternative cell-death pathways, including necrosis, pyroptosis, and ferroptosis [22]. On the other hand, as the primary detoxification and metabolic center in animals, the liver is a major target organ for Cr(VI) toxicity. A social health survey by Di Kangsu revealed a correlation between blood Cr levels in 305 Chinese individuals and liver injury marker enzymes, indicating that Cr(VI) can induce liver inflammation and damage [23]. Li’s laboratory investigations revealed that Cr(VI) induces rat liver injury via endoplasmic-reticulum-stress-mediated apoptosis [24]. Li et al. further demonstrated that low-dose Cr(VI) (0.05 mg/kg and 0.25 mg/kg K_2_Cr_2_O_7_) disrupts hepatic mitochondrial integrity in rats through the AMPK-dependent PINK1/Parkin signaling axis [25]. In Japanese medaka (*Oryzias latipes*), Li and colleagues showed that 46% of hepatic Cr ions were bound to metallothionein, underscoring the metal-binding response in liver cells [26]. Cr(VI) exposure also precipitates structural disintegration, inflammatory infiltration, and fibrotic remodeling in the hepatocytes of largemouth bass (*Micropterus salmoides*) and *Channa asiatica* [27,28]. Despite these advances, the impacts of Cr(VI) on oxidative status, bioaccumulation, trace-element homeostasis, and lipid metabolism in the liver of New Zealand rabbits remain undefined and warrant detailed investigation.

Metabolic-associated fatty liver disease (MAFLD) is characterized by aberrant lipid accumulation in the liver [29]. Hepatic lipid homeostasis depends on the dynamic balance among fatty acid uptake, β-oxidation, de novo lipogenesis, triglyceride synthesis and storage, and lipophagy. Disruption of this equilibrium results in hepatic steatosis that may evolve, via chronic inflammation and fibrosis, into progressive liver injury [30]. The endoplasmic reticulum (ER) and mitochondria are central hubs governing hepatic fatty acid metabolism. Key lipid-metabolic genes encode proteases that are anchored to the ER (e.g., *ELOVL6*, elongation of very-long-chain fatty acid protein 6; *DGAT1*, diacylglycerol O-acyltransferase 1; *PLINs*, perilipins; *ATF4*, cyclic AMP-dependent transcription factor 4; *PPARG*, peroxisome proliferator-activated receptor γ; seipin/Berardinelli-Seip congenital lipodystrophy 2 (*BSCL2*); and *FITM1*, fat-storage-inducing transmembrane protein 1) or reside in the nucleus, whereas apolipoprotein E (encode by *APOE*) and clusterin (encode by *CLU*) are cytoplasmic. Hepatocellular mitochondria adapt to metabolic cues, preventing triglyceride and lipotoxin accumulation; impairment of mitochondrial β-oxidation or oxidative phosphorylation provokes oxidative stress and is a defining feature of steatohepatitis [31]. Dysregulation in the expression of these lipid-metabolic genes precipitates disorders such as obesity and MAFLD. Current animal studies on Cr(VI)-induced hepatic lipid perturbations are restricted to rodents and poultry. Yang et al. demonstrated that chronic Cr(VI) exposure inhibits fatty acid synthesis and β-oxidation, aggravating hepatic dysfunction in Bufo gargarizans tadpoles [32]. Jia et al. reported altered hepatic mRNA expression of fatty acid synthase (FASN) and acyl-CoA oxidase 1 (ACOX1) in chickens exposed to Cr(VI) (K_2_Cr_2_O_7_) [33]. Nevertheless, the impact of Cr(VI) on lipid metabolism in New Zealand rabbit liver remains unexplored and warrants systematic investigation.

China is one of the world’s leading producers and consumers of rabbit meat. Among the breeds raised, the New Zealand rabbit is the most economically important and is extensively farmed nationwide. Valued for its rapid growth, prolific reproduction, and broad environmental adaptability, it has become the breed of choice for large-scale commercial operations. Beyond meat production, the New Zealand rabbit is widely employed as an experimental animal because its anatomy closely parallels that of humans. Nevertheless, the toxicological profile of Cr(VI) in this species remains largely unexplored [34,35]. In particular, the influence of Cr(VI) on hepatic lipid-metabolism genes has not yet been characterized. Existing studies on Cr(VI) toxicity in New Zealand rabbits have concentrated on gross morphological alterations, routine histopathological lesions, and reproductive toxicity [36,37,38]. The present investigation is the first to establish a juvenile New Zealand rabbit model of Cr(VI)-induced hepatic steatosis and to examine Cr(VI)-mediated liver injury, antioxidant gene expression, and lipid metabolism in this species.

## 2. Materials and Methods

### 2.1. Animal Treatment

**Figure 1:**
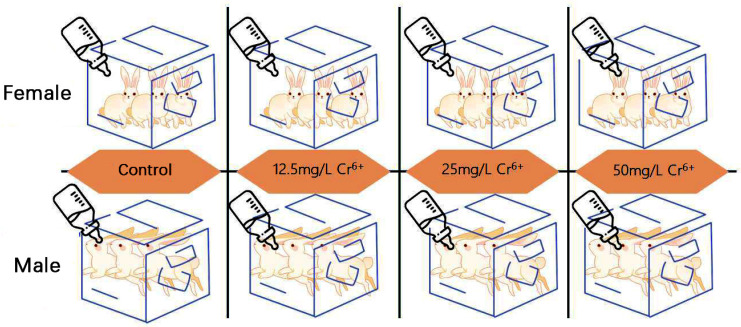
Schematic diagram of grouping.

Owing to the inherent limitations of cell experiments in accurately simulating animal blood circulation, inter-organ interactions, and overall physiological regulatory mechanisms, they are insufficient for comprehensively studying Cr(VI) poisoning. Conversely, the systemic effects of Cr(VI) exposure, such as the disruption of trace elements, accumulation of the Cr element, and alterations in blood cell levels observed in the rabbit liver, cannot be precisely replicated or assessed through cellular experiments alone. Consequently, we elected to utilize New Zealand rabbits as experimental subjects to investigate the systemic impacts of Cr(VI) poisoning. Twenty-four healthy New Zealand rabbits, 30 days old (479.0 ± 63 g), were obtained from a commercial farm in Yuanyang County, Henan Province, and housed in individually ventilated cages (IVCs) within the animal facility of the College of Veterinary Medicine, Henan Agricultural University. The IVC system features independent ventilation and air filtration, preventing cross-contamination and ensuring optimal air quality. Equipped with sensors for temperature, humidity, and airflow, it automatically adjusts these parameters in real time to meet the physiological needs of New Zealand rabbits. After adapting to the environment, the rabbits were divided into four groups using a stratified randomization method: a control group (fed with purified water) and three exposure groups, each receiving 12.5 mg/L, 25 mg/L, and 50 mg/L Cr (VI) (derived from potassium dichromate (K_2_Cr_2_O_7_)) in drinking water. In the grouping of New Zealand rabbits, a stratified randomization method was employed. Specifically, the rabbits were initially divided into two sub-strata based on gender, namely male and female rabbits. Subsequently, within each gender sub-stratum, further stratification was conducted according to the initial body weight of the rabbits. This approach ensured that the rabbits in each group were comparable in terms of the two key characteristics of body weight and gender. In selecting the concentrations of Cr (VI), reference was made to the reported levels in contaminated areas within China. For instance, Zhao Xiujun’s study revealed that the concentration of Cr (VI) in the groundwater of Jinchangbao near Jinzhou City, which is adjacent to a ferroalloy plant, reached 28.64 mg/L [39]. In addition, the research by Chen Wenfang indicated that the concentration of Cr (VI) in the groundwater at a contaminated site in Xinxiang City, Henan Province, was as high as 10 mg/L [40]. Therefore, in our study, after final weighing and consideration, we chose to add 25 mg/L of Cr (VI) to the drinking water of New Zealand white rabbits as the intermediate dose, while 12.5 mg/L and 50 mg/L were selected as the low and high doses, respectively, to establish animal models of Cr (VI) exposure in New Zealand white rabbits. Each group was subdivided into two cages (three rabbits per cage) to ensure adequate space (Figure 1). K_2_Cr_2_O_7_ (Tianjin Den Chemical Reagent Co., Ltd., Tianjin, China) was dissolved in purified water and supplied ad libitum; control animals received purified water only. The exposure period lasted 28 days. Cages and the rabbit room were disinfected every third day. All animals received a commercial fattening diet formulated to contain 16% crude protein, 2140 kcal/kg digestible energy, 22% crude fiber, 11.5% starch, 0.7% lysine, and 0.6% methionine + cysteine. The study was conducted in accordance with Chinese national guidelines for laboratory animal care and was approved by the Ethics Committee of Henan Agricultural University and the Ethics Committee of Henan College of Animal Husbandry and Economics (approval no. 202302001).

### 2.2. Sample Collection and Processing

Upon completion of the animal husbandry period, New Zealand rabbits were anesthetized with Rapid Sleep II and euthanized by rapid exsanguination and dissection after a 12 h fasting period. Whole blood was collected immediately and analyzed for physiological parameters using a fully automated veterinary hematology analyzer (BC-2800Vet, Mindray, Shenzhen, China). Intact livers were promptly harvested, rinsed with ice-cold phosphate-buffered saline (PBS, pH 7.4) to remove residual blood, and gently blotted dry with filter paper. When sampling, liver tissue samples are taken from five different liver lobules for preservation and analysis in a specific experiment to ensure the accuracy of the experimental results. Liver tissue was then sectioned into peanut-sized fragments for histological processing and TUNEL staining. Additional specimens were diced into mung-bean-sized cubes, fixed in electron microscopy fixative, and maintained at 4 °C for ultrastructural analysis. A portion of liver was transferred to a 5 mL centrifuge tube for chromium and trace element quantification. Another segment was placed in a 1.5 mL microcentrifuge tube and snap-frozen in liquid nitrogen for gene-level analyses. Peanut-sized liver samples from each group were immediately stored at −80 °C for subsequent Oil Red O staining.

### 2.3. Pathological Examination

Pathological changes in the liver tissue of New Zealand rabbits after Cr(VI) exposure were observed using hematoxylin and eosin (H&E) staining. The specific process of H&E staining included the steps as follows: firstly, taking fresh liver tissue and cutting it into tissue blocks of 1–2 mm^3^ size. The tissue blocks were immediately immersed in 4% neutral paraformaldehyde buffer solution and fixed at 4 °C for 48 h. Subsequently, the tissue underwent gradient ethanol dehydration (70% → 80% → 95% → anhydrous ethanol I → II, 1.5–2 h per stage) and xylene transparency (1 h each for I and II) to completely dehydrate and clarify the tissue. The transparent tissue blocks were then immersed in melted paraffin at 56–58 °C for three times (1–1.5 h each time) and embedded in a preheated paraffin mold to produce 5 μm thick sections. After dewaxing with xylene and gradient ethanol hydration, the sections were sequentially stained with hematoxylin (Harris staining solution for 8 min, hydrochloric acid alcohol differentiation for 10 s, followed by running water to restore blue) and eosin (0.5% eosin alcohol solution for 2 min), followed by gradient ethanol dehydration and xylene transparency. Finally, the sections were sealed with DPX neutral gum. A fluorescence microscope (Motic, AE31E, Xiamen, China) was used to observe the morphology and pathological characteristics of liver tissues, and high-resolution images were captured to annotate typical lesion areas.

### 2.4. Transmission Electron Microscopy Ultrastructure of Liver Tissue

Fresh liver tissue was rapidly diced into 1 mm^3^ cubes with a sterile surgical blade on an ultraclean workbench. The cubes were immediately immersed in ice-cold 2.5% glutaraldehyde electron-microscopy fixative (Zhengzhou Sevier Co., Ltd., Zhengzhou, China) and fixed at 4 °C for 12 h to preserve cellular ultrastructure and prevent autolysis. After fixation, samples were rinsed three times in 0.1 M PBS (pH 7.4), 15 min per rinse, then transferred to 1% osmium tetroxide (4 °C, dark) for an additional 2 h fixation. Subsequently, specimens were dehydrated in graded ethanol (30%, 50%, 70%, 80%, 90%, 95%, 10 min each; 100%, three changes, 15 min each), followed by substitution with propylene oxide. Tissue was then infiltrated with propylene oxide/Epon812 resin mixtures (3:1, 1:1, 1:3, 2 h each), soaked in pure resin overnight, and finally embedded in fresh resin. Polymerization was carried out at 60 °C for 48 h. Ultrathin sections (70 nm) were cut on a Leica UC7 ultramicrotome using a diamond knife and collected on 200-mesh copper grids. Sections were sequentially stained with 2% aqueous uranyl acetate (dark, 30 min) and freshly prepared Reynolds’ lead citrate (5 min), then examined with a Hitachi HT7800 transmission electron microscope (×3.0 K, 100 kv and Hybrid Contrast Mode) at the Yangzhou University Testing Center. A minimum of three representative micrographs were captured per sample.

### 2.5. TUNEL Staining

For tissue apoptosis staining, paraffin sections prepared in Assay 2.3 were deparaffinized in an environmentally friendly dewaxing agent (G1128) I, II, and III, 10 min each, rehydrated through absolute ethanol I, II, and III, 5 min each, and rinsed in distilled water. After air-drying, hydrophobic circles were drawn around the tissue with a PAP pen, and proteinase K working solution was applied within the circles and incubated at 37 °C for 20 min. Slides were rinsed three times in PBS (pH 7.4) on a rocking platform, 5 min per rinse. Sections were then covered with membrane permeabilization solution and incubated at room temperature for 20 min, followed by three PBS washes. Slides were incubated in 3% H_2_O_2_ at room temperature in the dark for 20 min and washed three times in PBS. After drying, equilibration buffer was applied within the circles and incubated at room temperature for 10 min. Reagent 1 (TdT) and Reagent 2 (dUTP) from the TUNEL kit were mixed with buffer at a 2:5:50 ratio, added to the circles, and incubated at 37 °C for 1 h in a humidified chamber. Slides were rinsed three times in PBS, then Streptavidin-HRP diluted 1:200 in TBST was applied and incubated at 37 °C for 30 min. Following three PBS washes, DAB chromogen was added dropwise to the circles, and color development was monitored microscopically; positive cells exhibited brownish-yellow nuclei. The reaction was stopped by rinsing with tap water. Sections were counterstained with hematoxylin, dehydrated, cleared, and finally imaged under a fluorescence microscope (Motic, Xiamen, China) to document hepatic apoptosis.

### 2.6. QRT-PCR Validation

Liver tissue (20 mg) was retrieved from liquid nitrogen and transferred into a 1.5 mL RNase-free EP tube. The tissue was minced and lysed with 1 mL TRIzol reagent (TIANGEN, Beijing, China), followed by vigorous vortexing to ensure complete homogenization. RNA extraction proceeded as follows: 0.2 mL chloroform was added, shaken vigorously for 10 s, and incubated at room temperature for 5 min. The mixture was then centrifuged at 12,000 rpm for 10 min at 4 °C in a refrigerated high-speed centrifuge. During centrifugation, fresh EP tubes were prepared to collect the aqueous phase. The colorless upper aqueous layer was carefully transferred to the new tubes. Subsequently, 0.5 mL isopropanol was added, gently inverted to mix, and incubated at room temperature for 10 min. The tubes were centrifuged again at 12,000 rpm for 10 min at 4 °C. The supernatant was discarded, and the pellet was washed with 1 mL 75% ethanol, followed by centrifugation at 12,000 rpm for 5 min at 4 °C. After removal of the ethanol, the pellet was air-dried under a laminar flow hood to obtain purified RNA. The dried RNA was dissolved in 50 µL RNase-free water. The purity and concentration of mRNA were determined using a UV spectrophotometer (NanoDrop, UL-1000, Wilmington, DE, USA) to normalize the mRNA concentration across different samples. Reverse transcription was performed using the FastKing cDNA First-Strand Synthesis Kit (TIANGEN, Beijing, China) to synthesize cDNA. Expression of lipid metabolism-related genes was quantified by qRT-PCR using the FastReal Rapid Fluorescence Quantitative PCR Premix (TIANGEN, Beijing, China) on a 7500FAST system (Singapore). Relative mRNA expression levels were calculated using 2^−ΔΔCT^ method [41,42]. All primers were synthesized by Sangon Biotech Co., Ltd. (Shanghai, China) based on sequences retrieved from https://www.ncbi.nlm.nih.gov (accessed on 5 June 2024). Primer names and sequences are listed in Table 1.

### 2.7. Oil Red O Staining

Remove the frozen section from the −80 °C refrigerator and restore it to room temperature. Fix it with tissue fixative for 15 min, wash it with tap water, and air dry it. The sections were immersed in the Oil Red O staining solution for 10 min (covered and protected from light). Background differentiation: The sections were briefly immersed in 60% isopropanol for 3 s, followed by a second immersion for 5 s, and then rinsed in two tanks of pure water for 10 s each. Hematoxylin counterstaining: The sections were immersed in hematoxylin counterstain for 5 min, washed in three tanks of pure water for 5 s, 10 s, and 30 s, respectively, differentiated in the differentiation solution for 8 s, and then washed with distilled water for 10 s each in two tanks. The sections were briefly immersed in tap water for 5 s and 10 s each, and the staining effect was examined under a microscope. Sealing: The sections were sealed with glycerol gelatin sealing agent. Microscopic examination, image acquisition, and analysis were performed.

### 2.8. Determination of Cr and Essential Trace Element Content in Tissue

A microwave digestion instrument (CEM, Mars6 Xpress, Matthews, NC, USA) was used to digest rabbit liver tissues and prepare the tissues into a solution form that can be measured by inductively coupled plasma optical emission spectroscopy (ICP-OES, Analytik Jena AG, Jena, German). The experimental steps for measuring elements in tissues are as follows: Tissue processing: Fresh liver tissues were cut into pieces and placed into 5 mL centrifuge tubes labeled with numbers. The test tube opening was placed in an oven and baked repeatedly at a temperature of 80 °C to remove moisture from the liver tissue. Grinding and weighing: After thoroughly drying the tissue, the liver tissues were ground in a mortar and accurately weighed 200 mg of the dry weight of the liver tissue sample. Digestion: The tissue samples were placed in the tissue digestion tubes in order, and 6 mL of concentrated nitric acid was added to each tube. The temperature and time settings for the microwave digestion instrument were as follows: 90 °C for 5 min, 130 °C for 5 min, and 185 °C for 25 min. Then the microwave digestion instrument was used to digest liver tissues and release several elemental solutions to be tested. Acid rush: The digestion inner tank was placed on the acid rush instrument, and the program was set (150 °C, 120 min) for acid rush to remove excess nitric acid from the solution. During the heating process of the acid rush machine, the solution content in the digestive tract was repeatedly observed. When the solution particles reached the size of mung bean particles, the acid rush operation was stopped. Constant volume: The acid-washed solution was transferred to a volumetric flask and diluted to the required 15 mL. The liquid samples with a constant volume will be used for experimental determination of element level.

### 2.9. Element Content Determination

After microwave digestion of liver tissue, the levels of chromium (Cr), iron (Fe), manganese (Mn), zinc (Zn), selenium (Se), and copper (Cu) elements in liver tissues were determined using ICP-OES. The element standard curve was set as follows: For Cu, the standard curve was set at 0, 0.5, 1, 2, 4, and 8 µg/mL; for Fe, the standard curve was set at 0, 1.5, 3, 6, 12, and 24 µg/mL; for Mn, the standard curve was set at 0, 0.05, 0.10, 0.2, 0.4, and 0.8 µg/mL; for Se, the standard curve was set at 0, 0.025, 0.05, 0.1, 0.2, and 0.4 µg/mL. A mixed standard solution was then prepared according to the standard curve to measure multiple elements simultaneously. The characteristic spectral lines of various elements were searched through https://www.foodmate.net/index.html (accessed on 20 April 2025), and the absorbance of the standard solution and the test sample were measured separately. The characteristic spectral lines of Cr, Cu, Fe, Mn, Se, and Zn elements are selected as 267.716 nm, 324.754 nm, 239.56 nm, 259.372 nm, 196.028 nm, and 206.200 nm, respectively. The level of Cr and several trace elements such as Fe, Mn, Zn, Se, and Cu were calculated by substituting the absorbance values into the standard curve. After converting the solution volume and sampling amount, the level of each element in the liver dry weight was obtained. Finally, GraphPad Prism software was used to calculate the distribution differences of each element in each group.

### 2.10. Statistical Analysis

Data underwent normality testing using the Shapiro–Wilk test. Following confirmation of normal distribution, differences among groups were analyzed using one-way ANOVA. The results were obtained using GraphPad Prism 9.0 software. The statistics of the number of lipid droplets in liver tissue during the experiment were jointly completed by ImageJ 1.48g and GraphPad Prism software. The results are expressed as the means ± standard deviations (SDs). Differences with a *p* value < 0.05 were considered statistically significant.

## 3. Results

### 3.1. Cr(VI) Downregulated Hematological Indicators in New Zealand Rabbits

**Figure 2:**
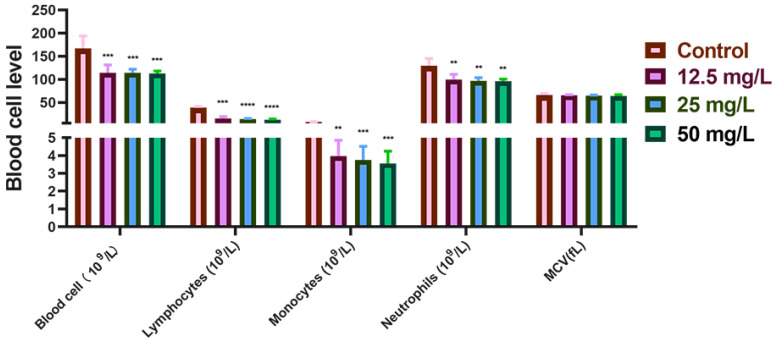
Use an automated blood analyzer to detect levels of several blood cells in whole blood. **: *p* < 0.01, ***: *p* < 0.001, ****: *p* < 0.0001.

First, the total blood cell count, neutrophil count, lymphocyte count, Monocyte count, and mean corpuscular volume (MCV) in the peripheral blood of New Zealand rabbits were determined. Relative to the control group, the total blood cell, neutrophil, Monocyte count, and lymphocyte counts exhibited either significant or non-significant reductions in a Cr(VI) concentration-dependent manner (*p* < 0.05, *p* < 0.01, *p* < 0.001, or *p* > 0.05). Specifically, MCV declined progressively with increasing Cr(VI) concentration, although this change did not reach statistical significance (*p* > 0.05) (Figure 2). Collectively, these hematological findings indicate that exposure to Cr(VI) at concentrations of 12.5 mg/L, 25 mg/L, and 50 mg/L exerts a discernible inhibitory effect on the blood cell indices of New Zealand rabbits compared with the control group.

### 3.2. Cr Accumulation and Pathological Damage in Liver Tissue

**Figure 3:**
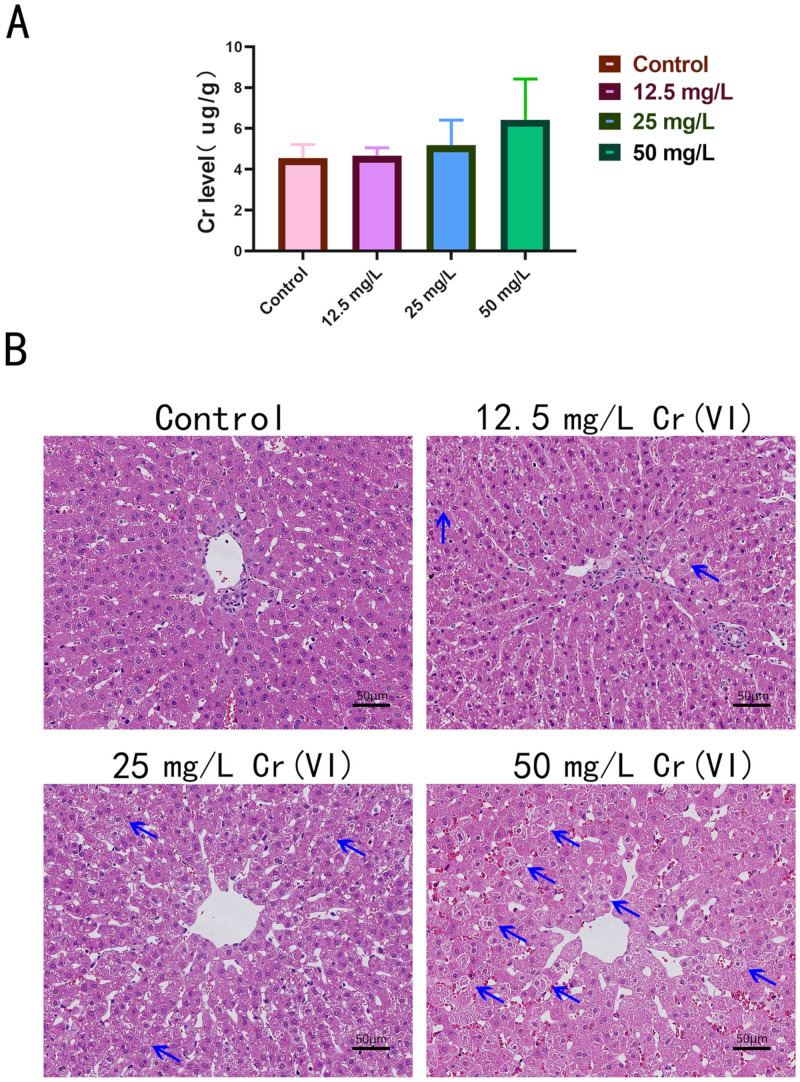
(**A**): ICP-OES detection of Cr level in rabbit liver tissue; (**B**): The pathological damage in liver tissue using HE staining (scale bar: 50 μm). The blue arrows in the figure represent liver cells that have been significantly damaged and have severely reduced cytoplasm.

Subsequently, hepatic Cr accumulation in New Zealand rabbits was quantified by ICP-OES. Our results revealed that hepatic Cr concentrations rose in parallel with increasing Cr(VI) exposure, exhibiting a clear dose–response relationship, although statistical significance was not attained (*p* > 0.05) (Figure 3A). The control group exhibited a baseline Cr level of 4.554 ± 0.66 µg/g, whereas the 50 mg/L Cr(VI) group achieved an accumulation of only 6.43 ± 1.9 µg/g. Histopathological evaluation of HE-stained hepatic sections further demonstrated that hepatocytes in the control group were structurally intact and displayed uniformly dark cytoplasmic staining. In the 12.5 mg/L and 25 mg/L Cr(VI) groups, intercellular vacuolation progressively intensified, hepatic cords broadened, and cytoplasmic staining became paler relative to controls. Administration of 50 mg/L Cr(VI) induced severe hepatic injury, manifested by interstitial disruption of hepatic cords, widened intercellular spaces, and extensive parenchymal cavitation. Moreover, numerous hepatocytes exhibited diminished cytoplasmic volume, lighter staining, and pronounced vacuolation (Figure 3B). Collectively, these findings indicate that supplementation of drinking water with 12.5, 25, and 50 mg/L Cr(VI) results in measurable hepatic Cr accumulation in New Zealand rabbits. Cr(VI) exposure elicits dose-dependent hepatotoxicity, with both Cr accumulation and the extent of hepatic damage increasing proportionally to the administered Cr(VI) concentration.

### 3.3. Trace Element Metabolism in Liver Tissue

Trace elements critically modulate hepatic steatosis by governing lipid metabolism, antioxidant defense, inflammatory responses, and cellular signaling pathways. Consequently, ICP-OES was employed to quantify selected essential trace elements in liver tissue. The data indicated that hepatic Mn, Se, and Fe concentrations progressively declined in a Cr(VI) concentration-dependent manner at 12.5, 25, and 50 mg/L. Relative to the control group, Fe levels diminished with rising Cr(VI) exposure (*p* < 0.05, *p* < 0.01 or *p* < 0.001), establishing a clear dose–response relationship with Cr(VI). In comparison with controls, hepatic Cu exhibited a modest elevation at 12.5 mg/L Cr(VI); nonetheless, this alteration was not statistically significant (*p* > 0.05). Cu concentrations peaked at 25 mg/L Cr(VI), yet the difference remained non-significant (*p* > 0.05), and subsequently declined at 50 mg/L, again without reaching significance (*p* > 0.05). Hepatic Zn displayed a slight, non-significant increase at 12.5 mg/L Cr(VI) (*p* > 0.05), whereas levels decreased at 25 mg/L and 50 mg/L, with no statistical significance (*p* > 0.05) (Table 2). Collectively, these results demonstrate that Cr(VI) exposure at the tested concentrations generally downregulates hepatic Zn, Fe, Mn, and Se, whereas Cu levels transiently rise from 12.5 mg/L to 25 mg/L Cr(VI) before declining from 25 mg/L to 50 mg/L Cr(VI).

### 3.4. Ultrastructural Changes in Liver Tissue

**Figure 4:**
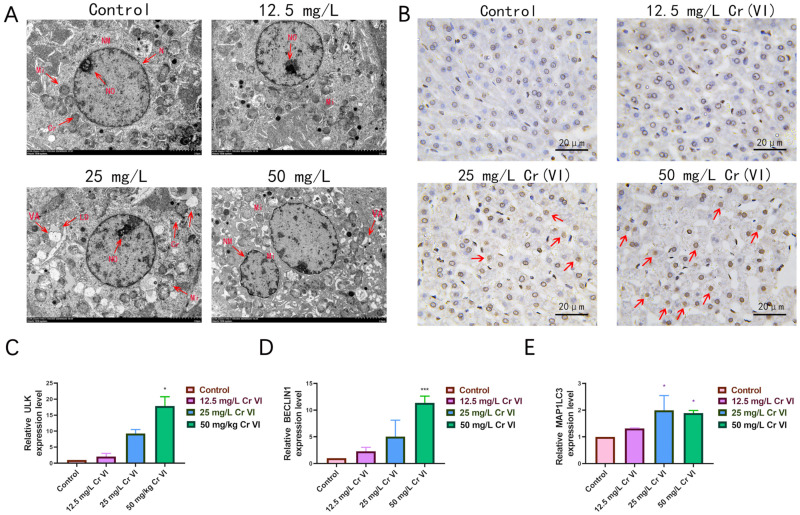
(**A**): Observation of mitochondrial damage and lipid droplets in liver tissue using transmission electron microscopy (scale bar: 2 μm). Mi: mitochondria, Sa: sarcomere, Cr: mitochondrial cristae, N: nucleus, NM: nuclear membrane, No: nucleolus, Ch: chromatin, VOs: vacuolar organelles, Aps: autophagosomes. (**B**): Cr(VI) induces apoptosis in liver tissue, with red arrows representing positive apoptotic nuclei. Scale bar: 20 μm. (**C**–**E**): The effect of Cr(VI) on autophagic genes expression. *: The difference was a significant difference (*p* < 0.05); ***: The difference was extremely significant (*p* < 0.001).

To further delineate Cr(VI)-induced hepatocellular injury, ultrastructural alterations in liver tissue under graded Cr(VI) exposures were examined by transmission electron microscopy (TEM). TEM observations revealed pronounced ultrastructural lesions, particularly in mitochondrial architecture, across all Cr(VI)-treated groups (Figure 4A). In the control cohort, mitochondria displayed normal morphology—ellipsoidal or rod-shaped profiles with intact, clearly delineated cristae. At 12.5 mg/L Cr(VI), mitochondrial morphology remained largely preserved, yet subtle changes emerged, manifesting as mild cristae indistinctness. At 25 mg/L Cr(VI), mitochondrial alterations became more evident: conspicuous swelling, irregular contours, and disordered cristae were observed. Intramitochondrial vacuoles appeared, and abundant cytoplasmic lipid droplets accumulated. The nucleus exhibited slight shrinkage. In the 50 mg/L Cr(VI) group, severe nuclear atrophy and extensive cytoplasmic vacuolation were evident. Mitochondria were markedly distorted, with some undergoing rupture; cristae were fragmented and markedly depleted, intramitochondrial vacuoles were numerous, and portions of the mitochondrial membranes were disrupted. Within apoptotic cells of the 50 mg/L Cr(VI) group, the endoplasmic reticulum cisternae dilated, forming small vesicles that fused with the plasma membrane and yielded distinctive surface blebs. In addition, in the 50 mg/L Cr (VI) group, we found a large number of double layered membrane structures surrounding mitochondria, which are speculated to be autophagosomes (Figure 4A). Complementary TUNEL staining corroborated significant hepatocellular apoptosis. Relative to controls, brownish-yellow nuclear positivity intensified in a Cr(VI) concentration-dependent manner, indicating progressive elevation of hepatic apoptosis (Figure 4B). Given the TEM-documented increase in vacuolar structures, we hypothesized that this change reflects elevated autophagosome formation. Consequently, qRT-PCR was employed to quantify the mRNA levels of key autophagy-related genes—*BECLIN1*, *ULK*, and *MAP1LC3*—in liver tissue. The data showed that *BECLIN1* and *ULK* expression rose progressively with increasing Cr(VI) concentration, whereas *MAP1LC3* expression increased from 0 to 25 mg/L and was marginally downregulated at 50 mg/L, yet remained significantly above control levels (Figure 4C–E). Collectively, these findings demonstrate that graded Cr(VI) exposures elicit ultrastructural damage, apoptosis, and autophagy in hepatic tissue.

### 3.5. Alterations in Antioxidant-Related Gene Expression

**Figure 5:**
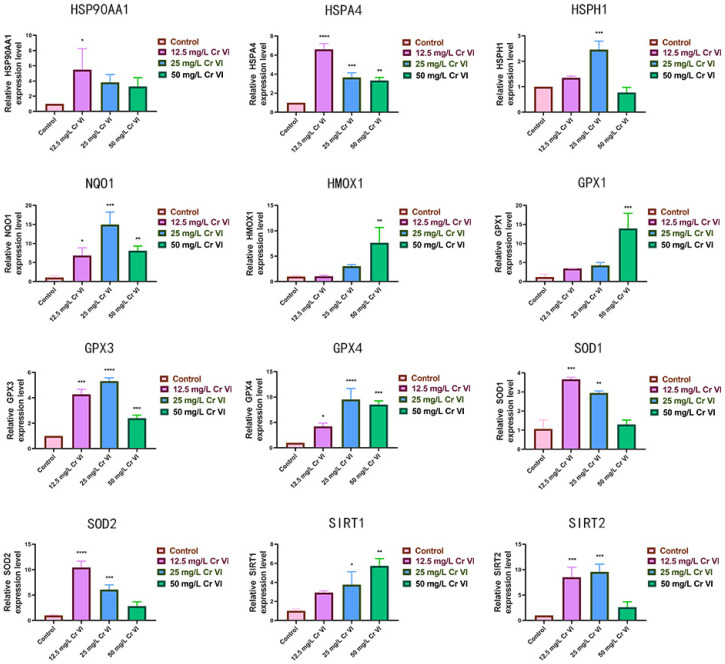
Changes in expression levels of key antioxidant genes in liver tissue. Compared to the control group. *: the difference was significant (*p* < 0.05); **: The difference was very significant (*p* < 0.01); ***: The difference was extremely significant (*p* < 0.001); ****: The difference was highly significant (*p* < 0.0001).

Cr(VI) exerts its principal toxicity through oxidative damage to animal cells; therefore, qRT-PCR was employed to quantify the hepatic expression of key antioxidant-related genes in rabbits exposed to graded Cr(VI) concentrations (Figure 5). Within the heat-shock protein family (*HSP90AA1*, *HSPA4*, *HSPH1*), the relative expression of *HSP90AA1* did not differ significantly from the control across Cr(VI) treatments (*p* > 0.05). Nevertheless, *HSP90AA1* expression rose progressively at 12.5 and 25 mg/L Cr(VI) and declined at 50 mg/L, while remaining above control values. Relative to controls, *HSPA4* expression differed markedly at 12.5, 25, and 50 mg/L Cr(VI) (*p* < 0.0001); it peaked at 12.5 mg/L, decreased at 25 mg/L, and rebounded at 50 mg/L. *HSPH1* expression increased at 12.5 mg/L Cr(VI) relative to the control (*p* < 0.001), continued to rise at 25 mg/L to its maximum level (*p* < 0.0001), and declined at 50 mg/L, albeit remaining significantly elevated (*p* < 0.05). Compared with the control, the antioxidant genes *HMOX1* and *NQO-1* exhibited up-regulation at 12.5 and 25 mg/L Cr(VI). At 50 mg/L Cr(VI), *NQO-1* expression was lower than at 25 mg/L but still above the control, whereas *HMOX1* expression surpassed the 25 mg/L level. The expression profiles of *SOD1* and *SOD2* were similar, peaking at 12.5 mg/L and decreasing thereafter, yet remaining consistently above control levels. *SIRT1* expression increased monotonically with Cr(VI) concentration. *SIRT2* expression was significantly higher than control at 12.5 and 25 mg/L Cr(VI) (*p* < 0.001) and declined sharply at 50 mg/L, though still exceeding control values. The *GPX1*, *GPX3*, and *GPX4* genes were up-regulated at 12.5 and 25 mg/L Cr(VI); at 50 mg/L Cr(VI), *GPX3* and *GPX4* expression fell relative to 25 mg/L yet remained above control levels. Collectively, these data indicate that, relative to the control, graded Cr(VI) exposures elicit a measurable activation of the antioxidant defense system in the liver of New Zealand rabbits.

### 3.6. Lipid Droplet Accumulation in Liver Tissue

**Figure 6:**
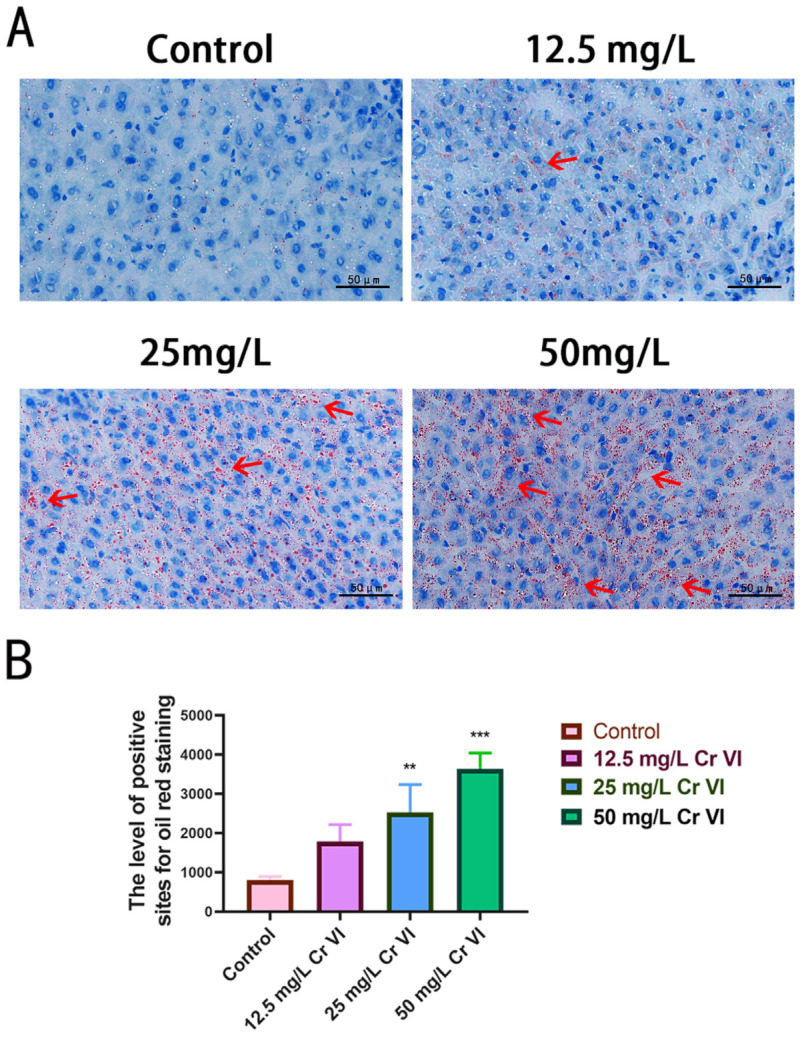
(**A**) Oil Red O staining illustrating lipid droplet structures in hepatic tissue (scale bar: 50 µm). The red arrow in the figure represents the positive lipid droplet structure. (**B**) ImageJ software was employed for automated quantification of lipid droplet number and diameter across multiple fields. A calibrated scale was applied to each micrograph, and the software subsequently counted red-stained lipid-positive foci in liver sections from the control group and the three Cr(VI) exposure groups (≥3 distinct stained regions per group). Inter-group differences were analyzed with GraphPad Prism. **: *p* < 0.01, ***: *p* < 0.001.

Histopathological sections suggested that 50 mg/L Cr(VI) induces obvious intracellular vacuolation, possibly reflecting hepatic lipid accumulation (Figure 3B). To verify this, Oil Red O staining of liver tissue was performed. Oil Red O-stained sections revealed that the control group displayed scant red-stained lipid droplets, accompanied by regular, uniformly sized hepatocyte nuclei, indicative of normal lipid metabolism and cellular architecture. In the 12.5 mg/L Cr(VI) group, discrete red lipid droplets emerged, signifying incipient dysregulation of lipid metabolism and initial intracellular lipid deposition. The 25 mg/L Cr(VI) group exhibited a marked increase in red lipid droplets and pronounced intracellular lipid accumulation (*p* < 0.01). At 50 mg/L Cr(VI), red lipid droplets were further augmented and more diffusely distributed, representing the most severe intracellular lipid accumulation observed (*p* < 0.001) (Figure 6A). Quantitative assessment of lipid droplet counts across multiple microscopic fields confirmed a dose-dependent relationship between lipid droplet formation and Cr(VI) concentration (Figure 6B). Collectively, these data demonstrate that, relative to controls, Cr(VI) at 12.5, 25, and 50 mg/L elicits hepatic lipid deposition, with the extent of lipid degeneration increasing proportionally to Cr(VI) concentration.

### 3.7. Changes in Lipid Metabolism-Related Gene Expression

**Figure 7:**
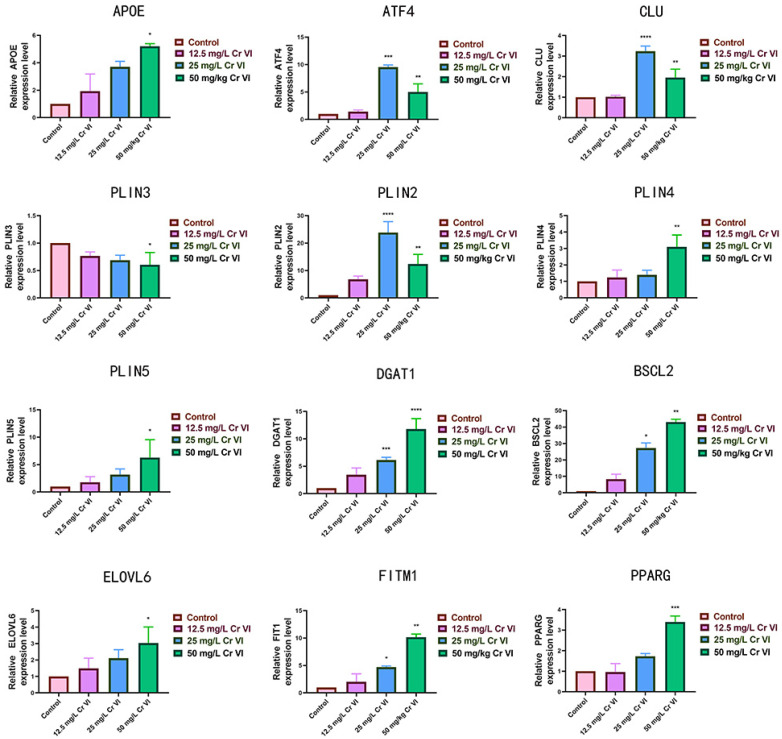
The effect of different concentrations of Cr (VI) on the expression of lipid metabolism related genes in the liver tissue of New Zealand rabbits detected by fluorescence quantitative PCR technology. Compared with the control group, * showed a significant difference (*p* < 0.05); **: The difference was very significant (*p* < 0.01); ***: The difference was extremely significant (*p* < 0.001); ****: The difference was highly significant (*p* < 0.0001).

The hallmark of lipid-metabolic disturbance is lipid-droplet formation, governed by genes controlling synthesis (*APOE*, *CLU*, *ELOVL6*, and *DGAT1*), intracellular transport and stability (*PLIN2–5*), and degradation (*ATF4*, *PPARG*, *BSCL2*, and *FITM1*) [43]. These genes are pivotal in hepatic lipid metabolism, and their expression and activity are precisely modulated by multiple signaling pathways. Therefore, their hepatic expression was quantified by qRT-PCR. Figure 7 illustrates the impact of graded Cr(VI) concentrations on lipid-metabolism gene expression in New Zealand rabbit liver. Comparing to the control group, we found exposure to Cr(VI) up-regulated *APOE*, *BSCL2*, *FITM1*, *PLIN2*, *PLIN4*, *PLIN5*, *PPARG*, and *ELOVL6* to varying degrees. *ULK* expression increased with Cr(VI) concentration (12.5–50 mg/L) (*p* > 0.05, *p* < 0.05 or *p* < 0.01), peaking at 50 mg/L (*p* < 0.01). *ATF4*, *PLIN2*, and *CLU* were markedly elevated at 25 mg/L (*p* > 0.05), but only marginally increased at 50 mg/L without significance (*p* < 0.01). *PLIN3* expression declined progressively with rising Cr(VI) concentration (*p* > 0.05, *p* < 0.05 or *p* < 0.01). These findings indicate that Cr(VI) at 12.5, 25, and 50 mg/L broadly elevates the expression of genes favoring lipid degeneration in New Zealand rabbit hepatocytes, except for the *PLIN3* gene.

## 4. Discussion

Cr(VI) is a heavy-metal oxyanion endowed with strong oxidizing properties and marked environmental persistence. The present study is the first to systematically confirm the impacts of Cr(VI) on defense mechanisms, trace-element metabolism, and hepatic lipid metabolism in New Zealand rabbits. The results demonstrate that Cr(VI) markedly lowers multiple blood physiological indices in New Zealand rabbits; supplementing drinking water with ascending Cr(VI) concentrations led to hepatic Cr accumulation that rose in a concentration-dependent fashion. Cr(VI) injured the liver of New Zealand rabbits, manifested by widened intercellular spaces and diminished cytoplasmic density. Moreover, Cr(VI) elicited a concentration-dependent accrual of lipid structures within hepatic tissue. Finally, Cr(VI) provoked ultrastructural hepatic damage, disrupted trace-element homeostasis, and up-regulated antioxidant- and lipid-metabolism-related genes. Our findings reveal that Cr(VI) activates defense mechanisms, induces hepatic apoptosis, and disturbs lipid metabolism in New Zealand rabbits. This study provides critical experimental data on the genetic mechanisms of Cr(VI)-induced fatty liver in New Zealand rabbits, changes in hepatic trace element metabolism, and the accumulation patterns and levels of Cr(VI) in the liver. These insights are essential for understanding the molecular toxicology of Cr(VI) and guiding strategies to protect liver health.

Hematological analyses showed that Cr(VI) reduced total leukocyte, monocyte, and lymphocyte counts, reflecting suppressed immunity in Cr(VI)-exposed New Zealand rabbits. Prior work indicates that Cr(VI) inhibits NET formation in rat peripheral blood and triggers neutrophil apoptosis via AMPK-pathway suppression [44]. The decline in blood parameters observed here is likely attributable to Cr(VI)-mediated inhibition of bone-marrow hematopoiesis in New Zealand rabbits. Cr(VI) may also exert a direct effect on these blood cells, thereby inducing apoptosis and ultimately resulting in a decline in the physiological parameters of rabbit blood [45,46].

Studies report that Cr accumulates in animal tissues because intracellular reduction of Cr(VI) to Cr(III) generates multidentate complexes with cellular proteins that resist excretion [47]. Even low Cr(VI) concentrations can injure the liver: Palabshaw et al. observed that 2 mg/L Cr(VI) is hepatotoxic to zebrafish, provoking significant hepatocyte apoptosis and altered elemental profiles [48]. Shen Jiayuan demonstrated that Cr(VI) induces murine hepatic injury through an NF-κB-driven inflammatory cascade [49]. I İpek Boşgelmez reported that intraperitoneal potassium chromate (20 mg Cr/kg) elevated lipid peroxidation in mouse liver and kidney, depressed non-protein sulfhydryl (NPSH) levels, and reduced catalase (CAT) and superoxide dismutase (SOD) activities, alongside pronounced tissue Cr accumulation [50]. Lei Zhao exposed Channa asiatica to 1.0 mg/L Cr(VI) for 28 days and documented a five-fold hepatic Cr increase. In our study, ICP-OES likewise showed concentration-dependent hepatic Cr accrual in New Zealand rabbits; nevertheless, even at 50 mg/L, the elevation was modest—only ~1.5-fold above baseline. Despite this limited accumulation, H & E staining revealed overt hepatic pathology: interstitial cells between hepatocyte cords disappeared, intercellular gaps widened, and vacuolar cavities appeared. Most notably, 50 mg/L Cr(VI) caused chromatin rarefaction consistent with vacuolar degeneration.

Trace elements are indispensable for antioxidant defense and cellular growth. Few studies have addressed Cr(VI)-induced trace-element dyshomeostasis in animals. Dana Kotizova observed that acute 24 h Cr(VI) exposure in rats up-regulated hepatic Zn, Cu, Fe, and Cr [51]. In the present work, hepatic Cu first increased then decreased across the Cr(VI) gradient, mirroring our earlier finding that heavy-metal toxicity promotes hepatic Cu retention [52]. Conversely, hepatic Se, Mn, Fe, and Zn declined dose-dependently. Cr(VI)-elicited oxidative stress likely perturbs the antioxidant machinery, thereby disturbing Cu handling. Cu resides at the catalytic center of Cu/Zn-SOD (SOD1) and participates in diverse physiological processes; under oxidative or mitochondrial stress, Cu may aberrantly accumulate and trigger cell death. High Cu^2+^ concentrations further exacerbate toxicity by activating apoptosis, paraptosis, pyroptosis, ferroptosis, and cuproptosis [53,54,55,56,57]. Cr(VI) poisoning may derange normal Cu trafficking, impair hepatocellular Cu export, and culminate in hepatic Cu overload. Compared with other elements, Fe exhibited the steepest decline: 12.5, 25, and 50 mg/L Cr(VI) markedly reduced hepatic Fe (*p* < 0.05 or *p* < 0.01), whereas reductions of Cu, Zn, Se, and Mn did not reach statistical significance. Competitive inhibition of Fe^2+^ transport by Cr(III) at divalent-metal transporters likely underpins the Fe depletion [58]. Mn, the catalytic core of mitochondrial Mn-SOD (SOD2), may be lost following Cr(VI)-induced damage. Se is essential for glutathione peroxidases (GPXs), thioredoxin reductases (TXNRD), and selenoprotein-P (SEPP1), orchestrating antioxidant defense, immunity, and thyroid-hormone metabolism [59,60,61,62]. The robust Cr(VI)-triggered oxidative stress up-regulated numerous antioxidant genes, thereby consuming Se and provoking its depletion. Zn, the second most abundant trace metal, governs growth, proliferation, and immunity; its drop may further compromise hepatic resilience. Future research should focus on elucidating why acute exposure to Cr(VI) can cause elevated levels of Cu element in animals tissues under specific conditions.

TEM analysis revealed that Cr(VI) markedly perturbed hepatic ultrastructure, with mitochondrial morphology being particularly affected. Progressive, dose-dependent mitochondrial swelling, cristae fragmentation, and matrix rarefaction furnished compelling ultrastructural evidence of Cr(VI)-induced hepatotoxicity and presaged mitochondrial dysfunction. TEM further demonstrated that exposure to 50 mg/L Cr(VI) evoked nuclear deformation and atrophy in New Zealand rabbit hepatocytes—morphological hallmarks of apoptosis. TUNEL staining corroborated these findings, revealing significant hepatocellular apoptosis following exposure to 50 mg/L Cr(VI). Many sudies found that environmental stress caused apoptosis in animals [63,64]. Research has demonstrated that Cr(VI) is capable of inducing apoptosis, a process that is closely associated with the mitochondrial and endoplasmic reticulum damage it causes. Furthermore, the endoplasmic reticulum damage can activate the relevant apoptotic pathways. QRT-PCR additionally demonstrated that escalating Cr(VI) concentrations elicited up-regulation of autophagy-related genes in hepatic tissue, a result concordant with TEM observations. When toxic substances provoke oxidative stress, mitochondrial impairment, apoptosis, and autophagy typically manifest concurrently, suggesting that oxidative damage initiates autophagic pathways that possess both pro-apoptotic and cytoprotective attributes [65,66]. In this study, the upregulation of autophagy-related genes suggests an accumulation of damaged organelles within the cells. Autophagy is triggered to eliminate the substantial number of impaired mitochondria that have emerged. The presence of numerous autophagic structures, characterized by double-membrane layers surrounding the mitochondria, as depicted in Figure 4A, corroborates this observation.

Heat-shock proteins (HSPs) are cytoplasmic and mitochondrial molecular chaperones that preserve protein conformation, assist nascent-peptide folding, regulate multi-protein complex assembly/disassembly, and mediate intracellular trafficking. By shielding cells from thermal and chemical insults, they enhance survival; aberrant HSP expression participates in NAFLD pathogenesis [67,68]. *HSP90AA1* encodes HSP90α, a chaperone critical for protein folding, stabilization, and transport. Under stress, HSP90α maintains native protein conformation and prevents aggregation, serving as a NAFLD biomarker [69,70]. Shao Wen-Weng reported that mitochondrial HSP60 promotes hepatic fatty acid oxidation while mitigating mitochondrial stress and inflammation via SIRT3 signaling [71,72]. In our experiment, Cr(VI) induced a broad yet moderate up-regulation of HSP genes, signifying activation of hepatoprotective responses to Cr(VI)-elicited oxidative stress. NQO1 and HMOX1 are pleiotropic antioxidants and anti-inflammatory cytoprotectants that modulate chromatin oxidative stress and DNA damage [73,74,75,76]. The GPX family comprises Se-dependent enzymes that scavenge hydroperoxides and participate in regulatory or biosynthetic pathways. GPX1, GPX3, and GPX4 inhibit H_2_O_2_- or lipid-peroxide-driven phosphorylation cascades, whereas GPX2 governs intestinal regeneration versus apoptosis and modulates carcinogenesis [77]. SOD1 and SOD2 detoxify superoxide anions to oxygen and hydrogen peroxide [78]. Sirtuins orchestrate cell-cycle progression, mitochondrial biogenesis, insulin secretion, redox balance, inflammation, and apoptosis [79]. Thus, Cr(VI) up-regulates antioxidant genes as a compensatory response to oxidative injury.

Under physiological conditions, dietary supplementation of Cr at appropriate levels exerts a cholesterol-lowering effect in cells [80]. Conversely, elevated concentrations of Cr(VI) may disrupt lipid homeostasis and precipitate fatty liver development. Our findings demonstrate that exposure to graded Cr(VI) concentrations induces hepatic steatosis. Oil Red O staining revealed a progressive, concentration-dependent accumulation of cytoplasmic lipid droplets. Specifically, as the Cr(VI) concentration rose from 12.5 mg/L to 50 mg/L, the number of red-stained lipid droplets in juvenile rabbit hepatocytes increased, accompanied by progressively severe lipid deposition. These data indicate that escalating Cr(VI) concentrations exacerbate intrahepatic lipid metabolic disturbances, inflicting increasingly pronounced interference on hepatic lipid metabolism and culminating in pathological manifestations such as hepatic steatosis.

Hepatic lipid-droplet formation involves the coordinated action of multiple genes. Cr(VI)-induced oxidative stress alters transcription of lipid-synthesis and droplet-maintenance genes. Yu Xuanpeng observed that 20 µmol/L K2Cr2O7 increased triglyceride (TG) and low-density lipoprotein cholesterol (LDL-C) in chicken LMH hepatoma cells, accompanied by up-regulation of ACACA and FASN [81]. Our qRT-PCR analyses revealed marked up-regulation of *APOE*, *DGAT1*, *PPARG*, *FITM1*, *BSCL2*, *CLU*, *ATF4*, and *ELOVL6* following Cr(VI)-induced steatosis, reflecting accelerated hepatic lipid turnover. APOE regulates hepatic lipoprotein uptake and fatty acid oxidation, maintaining systemic lipid balance [82,83]. DGAT1 catalyzes the synthesis of triglycerides and also regulates fat oxidation, metabolism, and energy balance. Moreover, an increase in DGAT1 activity can promote the formation of fatty liver, whereas inhibiting DGAT1 activity can protect the liver from the effects of fatty liver [84]. Perilipins 2–5 reside on the endoplasmic reticulum and modulate lipid-droplet dynamics [85]. Perilipin-3 tags nascent droplets and is later replaced by perilipin-2/5 during maturation [86]. Late-stage fatty liver down-regulates *PLIN3* as an adaptive response; our data confirmed a concentration-dependent *PLIN3* decrease. Perilipin-2 and Perilipin-3 shield droplet cores from lipases; Perilipin-2 is abundant in non-adipose tissues and correlates with steatosis—*PLIN2*-null mice exhibit lower hepatic TG and resist diet-induced obesity [87,88,89]. Perilipin-4, with extended amphipathic helices, coats droplets and rectifies phosphatidylcholine-deficient morphological defects [90]. Its rise here indicates Cr(VI)-stimulated lipid synthesis. *PLIN5* expression is ROS-responsive and regulates droplet turnover and fatty acid oxidation. PPAR-γ, a nuclear receptor, promotes fatty acid oxidation and lipogenesis; over-activation fosters steatosis [91,92,93]. PPAR-γ that encoded by *PPARG* gene controls fatty acid oxidation and storage; its ligand-activated transcriptional program is central to adipogenesis and glucose homeostasis. *ELOVL6* encodes an enzyme synthesizing long-chain fatty acids, directly impacting fatty acid synthesis rates. *BSCL2*, *FITM1*, *ATF4*, and *CLU* are key components of the autophagy initiation complex, participating in autophagy and potentially affecting lipid droplet degradation. *BSCL2* encodes seipin, a key protein for fat storage, with mutations causing lipid metabolism disorders [94]. Seipin is mainly involved in the formation and stability of lipid droplets, playing a crucial role in lipid droplet formation. Seipin combines with LDAF1 to define lipid droplet formation points in the endoplasmic reticulum, promoting the growth and maturation of small newborn lipid droplets into larger mature lipid droplets. Abnormal expression of the *BSCL2* gene is associated with the occurrence and development of steatosis [95]. *FITM1* encodes a protein involved in fatty acid binding and transport [96,97]. The *FITM1* gene plays an important role in lipid droplet formation, directly binding to diacylglycerols and triacylglycerols, participating in fatty acid transport and metabolism in the liver. Increased *FITM1* gene expression in this experiment indicates its impact on liver uptake and distribution of fatty acids, thereby affecting fatty degeneration processes [98]. ATF4 is a stress-responsive transcription factor modulating lipogenesis, fatty acid oxidation, lipoprotein metabolism, lipid storage, and inflammation; iron deficiency enhances ATF4-driven steatogenesis via HIF2α [99]. Clusterin influences lipid transport and apoptosis. Future studies should pinpoint the specific genes through which Cr(VI) precipitates fatty liver in New Zealand rabbits.

Of course, there are still some unresolved issues in our research. Through the application of qRT- PCR, we have undertaken a comprehensive analysis of pro-fatty liver genes in New Zealand rabbit liver tissue, demonstrating a robust approach to elucidating the genetic underpinnings of Cr(VI)-induced fatty liver. However, the precise major genes that are predominantly activated by Cr(VI) to trigger fatty liver development in New Zealand rabbits have yet to be conclusively identified. Moreover, our study has not fully addressed the mechanism behind the elevated Cu levels in liver tissue when exposed to Cr(VI) at concentrations of 12.5 mg/L and 25 mg/L. These areas present intriguing avenues for future investigation.

## 5. Conclusions

Graded Cr(VI) supplementation in drinking water produced hepatic Cr accumulation, trace-element imbalance, and structural injury in New Zealand rabbits. Cr(VI) dose-dependently increased hepatic lipid-droplet burden and up-regulated genes governing antioxidant defense and lipid metabolism, demonstrating activation of protective mechanisms yet disruption of lipid homeostasis. Our study provides an animal experimental model for investigating the effects of Cr(VI) on fatty liver metabolism in New Zealand rabbits. Future research should focus more on studying the specific gene expression changes or protein pathways induced by Cr(VI) that lead to metabolic disorders in fatty liver of New Zealand rabbits.

## Figures and Tables

**Table 1:** Positive and Negative Primer Sequence for Gene Detection.

Gene	Forward Primer	Reverse Primer
*APOE*	GCAGGAGGCGACTGACC	GCAGGTAATCCCAGAAGCGG
*BSCL2*	AGTCACAAGTGACAGAGCCAG	CTGGCGGCTCCTCTTCCT
*FITM1*	TTGCAAAAACGGTGCTGAACTTAT	ATGTGCTGGTCACCCTTCAC
*PLIN3*	GCCGCAGATTGCGTCG	CCGACACCTTGGATGACACA
*PLIN4*	GTCAGTGGAGGAGTGTGGTC	ACTGCCAGCTGAGCTTGTTC
*PLIN5*	CTCAACTTTCCTGCCCGTCA	CTGATCACCGGACATTCTGCT
*PLIN2*	AGTGTCCGACAGCTTCCTCA	CAGTGAAATCAAATGCCAGCCAG
*PPARG*	GGTGGTTGAGAGCAGTGGAT	CTGTGTCAACCATGATGACTTCTTT
*DGAT1*	CCACTGGGAGCTGAGATGC	CCAGGAACAACCGTGCATTG
*CLU*	CGTGGAGTTCATCACAGGAGG	TGGCACTTAGCACACTGGTC
*HSP90AA1*	GCCCAGAGTGCTGAATACCC	TAACAGGTGCCCTGCTTCTC
*HSPA4*	TCCAGTGCCTCTCTAGTGGA	AGCTTGAGAAGTCACGCCG
*HSPD1*	GTAAGCCCCTGGTCATAATTGCT	TCCCTCTTCTCCAAATACTGCAC
*SOD1*	CACCATCCACTTCGAGCAGA	CTGCACTCGTACAGCCTTGT
*SOD2*	GCAAGGAACAACAGGCCTTA	AACAGCCAGAGAGCACGAC
*HMOX1*	GCCGAGGGTTTTAAGCTGGT	CAGCTCCTCCGGGAAGTAGA
*NQO1*	CTTCAACCCCGTCCTTTCCA	ACTGCAGCGGGAACTGAAATA
*SIRT1*	TAACTGGAGCTGGGGTGTCT	GACGGTTGAAACTGTCCAGG
*SIRT2*	GGTGCAAGAGGCTCAGGATT	AGCGGTCGCTCTGAATGAAG
*GPX1*	AACCAGTTTGGGCATCAGGAGA	GGCCTTGGCGCCGTT
*GPX3*	GGGGCCAAGAGAAGTCCAA	TCTTGTAGTGCATTCAGTTCCACG
*GPX4*	CGGGAGGCAGGAGCC	GGTGAAGTTCCACTTGATGGC
*MAP1LC3*	GGATCACAGTCCCTTCCTCG	CTTACAACGGTCGGCAAAGC
*BECLIN1*	GCCGAAGACTGAAGGTCACT	ACGTTGAGCTGAGTGTCCAG
*ULK*	ACCGTGGGCAAGTTCGAG	ACCGTGGGCAAGTTCGAG
*Actin*	CAGTGGCCGTACAACTGGTAT	AAACGCAAGATCGCATGTGG

**Table 2:** Trace element level in liver tissue.

	Cu (μg/g)	Zn (μg/g)	Fe (μg/g)	Mn (μg/g)	Se (μg/g)
Control	1.79 ± 0.588	15.29 ± 1.326	1061 ± 226.7	250.4 ± 17.32	3.981 ± 0.262
12.5 mg/L	1.82 ± 0.599	15.47 ± 1.896	641.3 ± 57.38 (*)	247.5 ± 25.86	3.938 ± 0.324
25 mg/L	2.56 ± 0.417	14.39 ± 0.015	517.4 ± 140.9 (**)	219.6 ± 27.25	3.661 ± 0.217
50 mg/L	1.51 ± 0.199	14.35 ± 1.154	464.6 ± 142.6 (***)	214.6 ± 23.89	3.642 ± 0.322

Detection of several essential trace element levels in liver tissue using ICP-OES. Compared with the control group, *: The difference was significant difference (*p* < 0.05); **: The difference was very significant (*p* < 0.01); ***: The difference was extremely significant (*p* < 0.001).

## Data Availability

According to reasonable requirements, data can be obtained from the first author.

## Abbreviations

The following abbreviations are used in this manuscript:

Cr	Chromium
Cr(III)	Trivalent chromium
Cr(VI)	Hexavalent chromium
ROS	Reactive oxygen species
ER	Endoplasmic reticulum
ATF4	Cyclic AMP-dependent transcription factor 4
FASN	Fatty acid synthase
ACOX1	Acyl-CoA oxidase 1
BSCL2	Berardinelli-Seip congenital lipodystrophy 2
NCBI	National Center for Biotechnology Information
IVC	individually ventilated cages
TEM	Transmission electron microscopy
SOD1	Superoxide dismutase 1
GPX	Glutathione peroxidase
HSPs	Heat shock proteins
APOE	Apolipoprotein E
CLU	Clusterin
PPARG	Peroxisome Proliferator Activated Receptor Gamma
ELOVL6	Elongation of Very Long Chain Fatty Acids Protein 6
DGAT1	Diacylglycerol O-Acyltransferase 1
PLINs	Perilipins
FITM1	Fat storage inducing transmembrane protein 1
MAFLD	Metabolic-associated fatty liver disease
Fe	iron
Mn	Manganese
Zn	Zinc
Se	Selenium
Cu	Copper
ICP-OES	Inductively coupled plasma optical emission spectroscopy
TXNRD	thioredoxin reductases
SEPP1	selenoprotein-P
TG	Triglyceride
LDL-C	Low-density lipoprotein cholesterol
